# A submersible battery-powered flow injection (FI) sensor for the determination of nitrate in estuarine and coastal waters

**DOI:** 10.1155/S1463924699000012

**Published:** 1999

**Authors:** Anthony R. J. David, Trevor McCormack, Paul J. Worsfold

**Affiliations:** Department of Environmental Sciences Plymouth Environmental Research Centre University of Plymouth Plymouth PL4 8AA UK

## Abstract

The design, construction and performance of a remotely deployed submersible flow injection-based nutrient (total oxidized nitrogen) sensor are described. The sensor featured a custom-built microcomputer and a solid-state, flow-through spectrophotometric detector, and the derivatization chemistry was based on in-line coppercadmium reduction of nitrate to nitrite, and diazotization with N1NED and sulphanilamide. The limit of detection was 0.0014 mg l^-1^ NO_3_-N and the linear range was 0.0014- 0.77 mg l^-1^ with a 260 μl sample volume and a 20 mm path length flow cell. Results from submersed deployments in the Tamar estuary and North Sea are also reported.


Journal of Automated Methods & Management in Chemistry, Vol. 21, No. 1 (January–February 1999) pp. 1–9

### A submersible battery-powered flow injection (FI) sensor for the determination of nitrate in estuarine and coastal waters

**Anthony R. J. David, Trevor McCormack and Paul J. Worsfold**

*Department of Environmental Sciences, Plymouth Environmental Research Centre, University of Plymouth, Plymouth PL4 8AA, UK*

*The design, construction and performance of a remotely deployed submersible flow injection-based nutrient (total oxidized nitrogen) sensor are described. The sensor featured a custom-built microcomputer and a solid-state, flow-through spectrophotometric detector, and the derivatization chemistry was based on in-line copper–cadmium reduction of nitrate to nitrite, and diazotization with N1NED and sulphanilamide. The limit of detection was 0.0014 mg $l^{-1}$ $NO_3$–N and the linear range was 0.0014–0.77 mg $l^{-1}$ with a 260 μl sample volume and a 20 mm path length flow cell. Results from submersed deployments in the Tamar estuary and North Sea are also reported.*

### Introduction

Nitrate is a growth-limiting micronutrient in the euphotic surface layers of sea water, where light can penetrate, and its concentration in coastal waters is regulated by advective transport of nitrate to the surface layers and assimilation by organisms, e.g. phytoplankton. This gives rise to seasonal trends in nitrate concentrations in coastal waters. Winter cooling within the water column causes vertical mixing, thereby allowing the upwelling of nutrients to enrich the surface layers, whereas in late spring and summer, nitrate concentrations are depleted by increased biological activity [1].

The level of nitrate in freshwater systems has increased significantly in the past 50 years and this is attributable to more intensive agricultural practices utilizing nitrogenous fertilisers. Riverine nitrate concentrations also follow a definite seasonal pattern that is related to the flow of the river [2,3]. Plant uptake of nutrients in soils during the growing season keeps nitrate concentrations low despite the addition of nitrate-containing fertilisers. Maximum transpiration and evaporation also occurs during this period, which reduces the amount of water in the soil capable of leaching out nitrate. When the growing season has ended, nitrate concentrations in river systems increase, corresponding to the time when higher concentrations of nitrate are found in coastal waters.

The influx of nitrate from freshwater systems contributes to the overall marine nitrogen cycle, and this effect is at a maximum where estuarine and coastal waters merge. There is a fine balance between sufficient nutrients to promote healthy but controlled growth of micro-organisms (mesotrophic status), and excess levels, which are likely to cause excessive algal blooms (eutrophic to hypertrophic status); a situation that can cause stress to organisms higher up the food chain. In autumn this can also give rise to the proliferation of cyanobacteria. Therefore, careful management and control of nutrient concentrations is required, for which accurate and reliable monitoring techniques are essential.

Currently, nitrate concentrations are measured by collecting discrete samples in the field or onboard ship, and analysing them at the collection site (if convenient) or after transportation to a central laboratory. Apart from the cost and logistical problems of monitoring a particular area using this approach, sample preservation techniques can affect sample integrity, e.g. freezing of coastal and estuarine waters at $-10\,°C$ gave a small decrease in observed nitrate concentrations [4], whereas freezing at $-20\,°C$ gave more reliable results [5]. The US EPA methodology for the determination of anions in water states that unpreserved samples must be analysed within 48 h or be preserved with sulphuric acid at $pH < 2$ and stored for a maximum of 28 days [6]. However, a sample holding time of 28 days at low pH has been reported to convert nitrite to nitrate by microbial activity, whereas storing at a high pH had no adverse effect [7]. Therefore, field-based techniques are the most cost-effective way of continuously monitoring natural waters and eliminating any deleterious effects caused by sample preservation techniques.

Flow Injection (FI) is ideally suited to the automation of standard laboratory methods and has been successfully applied to process control [8] and environmental monitoring [9, 10]. It is now accepted by many organizations, e.g. US EPA, as a reference laboratory method for which instrumentation is readily available [11, 12]. Remotely deployed, FI-based field monitors have been developed for monitoring nitrate [13–16], phosphate [17], ammonia [18] and aluminium [19] in freshwaters. Submersible FI-based sensors have also been developed to monitor silicate, sulphide and nitrate in deep oceanic waters [20, 21].

This paper describes the construction and field deployment of a battery-powered submersible nitrate sensor, comprising an in-house designed microcomputer and solid-state spectrophotometric detector. All operations were automated for remote deployments over complete tidal cycles at depths of up to 30 m in coastal and estuarine waters.

1463-9246/99 $12·00 © 1999 Taylor & Francis Ltd

### Experimental

### *Reagents*

All solutions were prepared from ultra-pure water produced by a 'Milli-Q' system (Millipore Corporation), and all reagents were AnalaR® (BDH Merck) unless otherwise stated. The carrier solution was prepared by dissolving 10.0 g of ammonium chloride in 1 l of water. The N-(1-naphthyl)ethylenediamine dihydrochloride (N1NED, Sigma Chemicals) and sulphanilamide reagents were prepared by dissolving 0.5 g and 25.0 g, respectively, in 1 l of water containing 10% ($^v/_v$) orthophosphoric acid. The mixed colour reagent was prepared immediately before use by mixing equal volumes of the N1NED and sulphanilamide reagents in a brown glass bottle. A stock 100 mg $l^{-1}$ Nitrate-N solution was prepared by dissolving 0.7220 g of potassium nitrate in 1 l of water. Working standards were prepared by serial dilution of the stock solution with water immediately before use.

### *Instrumentation and procedures*

### *FI manifold*

The FI manifold was housed in an air-filled pressure vessel (maintained at atmospheric pressure) with reagents and sample pumped in and waste pumped out. This approach enabled the use of commercially available FI components, e.g. peristaltic pumps, switching and injection valves. It was preferred to the more commonly used pressure-compensated design in which the analyser compartment is filled with, e.g. oil maintained at ambient pressure. The FI manifold was chassis mounted and completely removable from the instrument pressure housing for maintenance and adjustment. This also allowed it to be used as a bench instrument and a terrestrial remotely deployed sensor. The manifold, shown in figure 1, had two 12 V DC peristaltic pumps (Ismatec) which delivered carrier solution, mixed colour reagent and sample/standards via switching valves and a modified six-port sample injection valve. A sample injection volume of 260 μl was used unless otherwise stated. The reduction column, reaction coil and solid state flowcells were specially developed for the sensor. The reagents were prepared in collapsible reagent bags and placed in the reagent module, which was open to the surrounding water. This kept the reagents at ambient pressure and temperature, assisted pumping and maintained the reaction chemistry at a stable temperature.

### *Pump tubing*

To satisfy concern regarding the long-term reliability of a peristaltic pump-based system, trials under controlled conditions were carried out on four types of pump tubing selected on the basis of manufacturers data for life expectancy under the experimental conditions used. A test rig was built and a test regime devised to evaluate the selected materials under controlled conditions. The four different tubing materials selected for comparison were: flexible modified PVC ('Tygon'), flexible PVC ('Tygon yellow'), silicone rubber and butyl rubber ('Ismaprene'). Each tube was sectioned using a specially constructed cutter to examine the bore profile and measure tube dimensions using a shadowgraph. The tubes were then subjected to hardness tests to compare grades of material supplied.

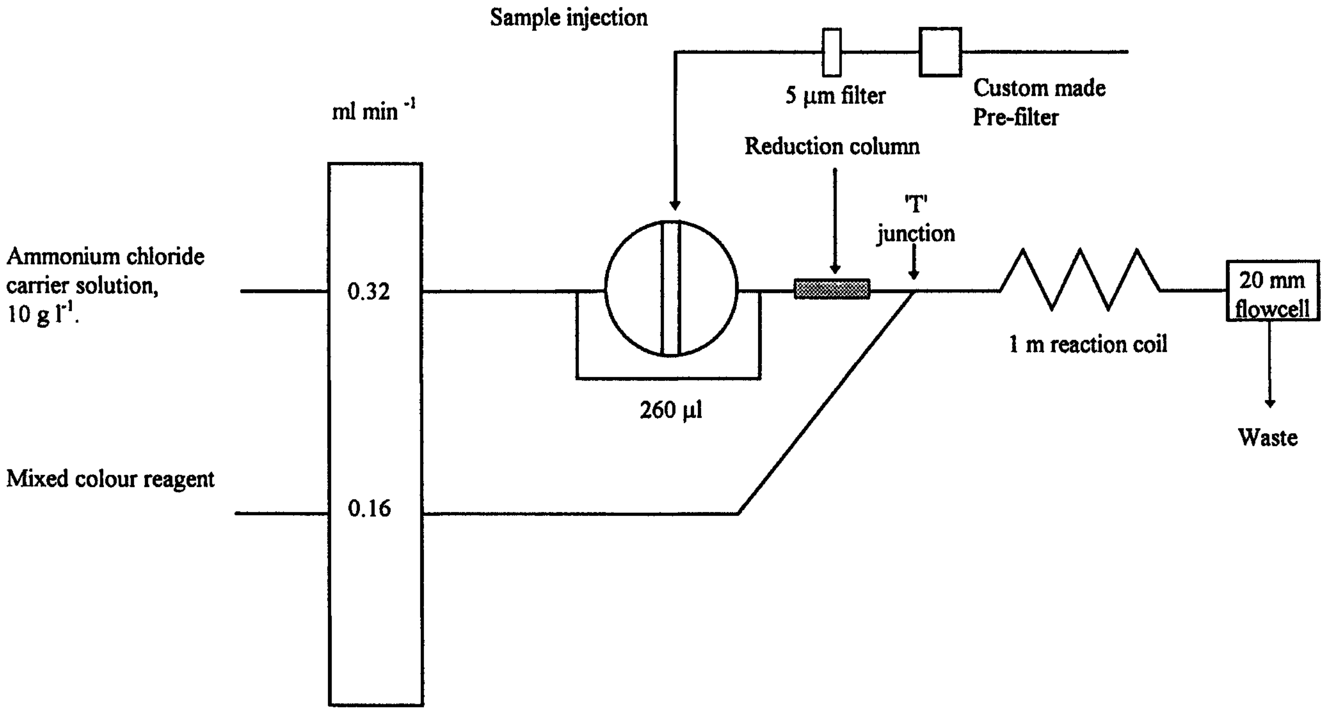

*Figure 1. FI manifold configuration for the determination of nitrate.*

*Reduction column*

For most of the bench testing of the FI manifold, copperized-cadmium packed reduction columns [22] were used. These were prepared by adding 50 ml of 10 $g l^{-1}$ copper sulphate solution to 5 g of 100 mesh (0.15 mm o.d.) cadmium powder and stirring for 2 min. The copperized-cadmium was then filtered off and washed with 2 M hydrochloric acid followed by ammonium chloride solution (10 $g l^{-1}$). Finally, the washed copperized-cadmium was packed into a $40 \times 2$ mm i. d. glass tube plugged at each end with clean glass wool. Inlet and outlet connections were simple push fits using PVC tubing. For submersed deployments, and to overcome the problems associated with packed reduction columns, e.g. trapped air, the column was redesigned and ruggedized. A prototype column with the same internal dimensions as the glass column was machined from clear acrylic rod. Each end was then counter-drilled and threaded to accept standard ¼ inch FI flange connectors. 'Nylon' mesh retainers and stainless steel washers were placed under the flange connectors to hold the copperized-cadmium in place.

*Flowcell*

Ease of maintenance demands that components of a field instrument are simple to service or replace *in situ*, and therefore a disposable solid-state flowcell was considered essential for this application. Prototype Z-cell configuration [23] flowcells incorporating an ultrabright green LED (Radio Spares No. 590-496) as the source, and a photodiode with peak response at 560 nm (RS No. 303-719) as the detector, were manufactured from clear acrylic rod to assist manufacture and observe liquid flow, before being painted black to eliminate the effects of stray light. A small aluminium heat sink was incorporated to eliminate any localized heating of the reaction mixture in the flowcell. Evaluation of the prototype flowcells was carried out using a small FI test rig and a series of nitrite standards (i.e. without the reduction column). Response from the photodiode was measured using a small custom-built amplifier connected to a millivoltmeter. A constant current 5 V supply to the LED was provided by including a 120 Ω series resistor in the circuit.

*On-board computer system*

The on-board control system was a development of that used in a terrestrial nutrient monitor developed at the University of Plymouth [15]. Several design changes were incorporated to improve performance, system and component reliability, and power consumption, and reduce the overall size. The on-board computer incorporated the well-proven Intel® 80C32 microprocessor, an MCS®- BASIC v 1.1 interpreter and an external 8 K EPROM. This configuration provided the functions essential for automatic control systems, i.e. BASIC programmable, EPROM programmable, internal clock auto boot on power up, floating point arithmetic, and internal and external interrupts.

The processor board was connected to the I/O board by 40-way edge connectors and ribbon cable. This board contained a central 82C55 peripheral interface adapter

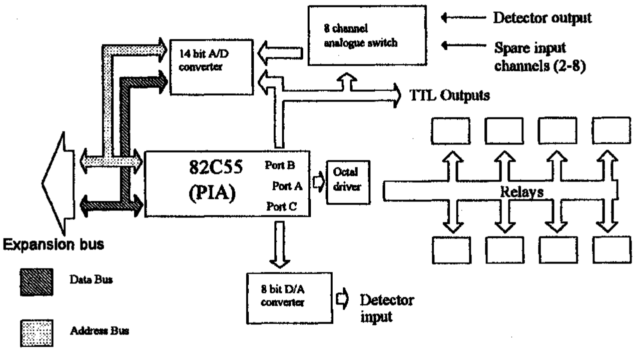

*Figure 2. Schematic diagram of the control system I/O board.*

(PIA), configured as three 8-bit TTL output ports. Port A was connected to a network of eight 12 V DC supply relays to control the peristaltic pumps, switching valve and injection valve. Port B provided TTL signals to a 14-bit analogue to digital converter (ADC), an eight channel analogue switch, and the injection valve to toggle between fill and inject positions. Port C provided the input to the 8-bit digital to analogue converter. A schematic representation of the I/O board is shown in figure 2.

*BASIC* control programmes were executed from the external *EPROM*, which could be modified on-line by transferring *ROM* to the 80C32s internal *RAM* using the *XFER* command. The on-board computer and control boards were finished to military specifications to provide a high level of reliability [24]. All circuit boards were cleaned to remove solder flux residues, and other organic and inorganic residues before conformally coating with a single-part acrylic-based conformal coating [25, 26]. Acrylic coatings provided a reasonable compromise between moisture resistance, and ease of application and handling. After conformal coating, the most vulnerable components were supported by applying a non-corrosive silicone RTV (room temperature vulcanizing) compound between the component and circuit board to prevent damage to component legs through vibration or shock. Non-corrosive RTV compounds cure with the liberation of alcohol, whereas most normal RTV compounds cure with the liberation of acetic acid, which can have deleterious effects on circuit boards.

*Sensor pressure housing*

A modular construction, i.e. separate FI manifold, filtration, reagent and power modules, as shown in figure 3, was used for the sensor in order to allow different specification components to be selected for different deployment scenarios, e.g. an estuarine deployment may require an upgraded filtration module to cope with the high level of suspended particulate matter, whereas deployment alongside other devices on a test platform with power supplied by the host system would negate the need for a power module.

The instrument pressure housing was constructed from 250 mm o.d. rigid PVC tubing with a wall thickness of 13 mm. Two flanged end caps were machined from 25 mm rigid PVC sheet; the bottom end cap being permanently bonded to the PVC tubing. A piston seal

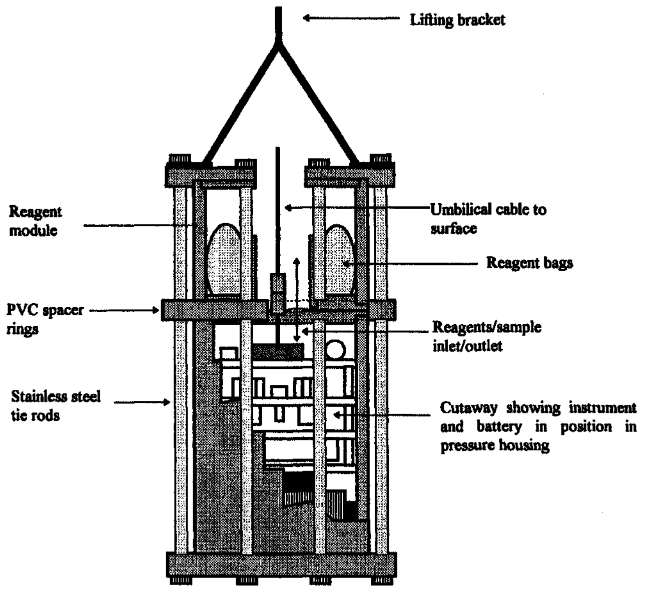

*Figure 3. Schematic diagram showing the configuration of the integrated sensor system used for submersed deployments.*

arrangement was incorporated into the upper and removable end cap to provide a sea seal which was subsequently tested to a depth of 45 m. An eight-way waterproof socket was fitted to the removable end cap to allow direct serial communication with a PC, external power and switching if required. A 50 m eight-core umbilical cable was provided for direct communications and control which terminated at the sensor end with the corresponding waterproof eight-way plug. The control end consisted of an interface box which housed the on/off switch for the monitor, indicators to show the position of the injection valve and a serial communication lead to connect to an external computer.

During submersed trials, the instrument was configured to run on internal batteries with the capability of being switched on/off remotely. This allowed the system to be immediately shut down if any anomalies were detected during real-time operation and therefore minimize damage should a leak occur. The instrument housing was capable of storing sufficient lead-acid batteries for seven days continuous deployment. A separate battery module capable of powering the system for 30 days was also constructed but not deployed. A specially designed tubing adapter, as shown in figure 4, was also constructed and fitted to the removable end cap to allow reagents, sample/standards and waste to be pumped between the ambient and atmospheric pressure compartments.

The various modules were held in position by an outer cage assembly (figure 3). This consisted of two end caps and an intermediate spacer ring machined from solid 35-mm-thick rigid PVC. Six lengths of M12 stainless steel studding formed the tie rods over which tubular stainless steel spacers were fitted to correctly position the PVC end caps and middle spacer ring. The stainless steel lifting bracket was located on two of the tie rod ends and the whole assembly was held together with M12 stainless steel 'Nyloc' locking nuts.

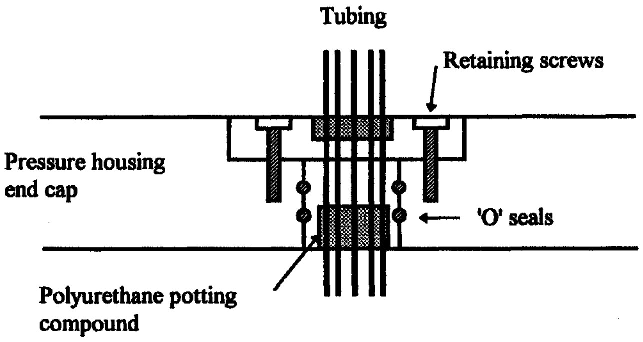

*Figure 4. Cross-section of custom-built circular tubing inlet/outlet device.*

This arrangement provided a method of holding the modules together without attaching fixings to the module walls, which would have created stress points; particularly in cold conditions when PVC is more susceptible to impact damage. The cage assembly also provided substantial protection to the sensor and attachment points for lifting brackets. For simple drop deployments from a research vessel, a single bracket was attached to the top of the cage assembly. For tethered buoy deployments, brackets could be attached to both ends of the cage assembly so that the sensor fits between the buoy and its anchor.

### Results and discussion

### *System chemistry*

Determination of nitrate plus nitrite, i.e. total oxidized nitrogen (TON) is routinely carried out by various spectrophotometric methods. Direct methods are based on the inherent UV absorbance of the nitrate ion [27, 28] or derivatization of the nitrate ion and detection in the visible region [29]. Indirect spectrophotometric methods require initial reduction of nitrate to nitrite by homogeneous [30, 31] or heterogeneous [32–40] reduction methods using reduction columns of powdered or granular metals, e.g. zinc [32, 33], amalgamated zinc [34], cadmium [35], amalgamated cadmium [36, 37] and copperized-cadmium [38–40]. A comprehensive optimization of the conditions for reduction using cadmium powder or granules was reported by Nydahl [41]. Copperized-cadmium wire [42, 43] and copperized-cadmium–silver wire [44, 45] (95% Cd) have also been used. Hydes and Hill [46] described the use of a copperized granulated 50:50 cadmium–copper alloy reduction column, and van Staden [47] reported the use of a copper tube pre-column followed by a copperized-cadmium tube reduction column. The use of copperized-cadmium is therefore a well-established method for nitrate reduction and forms the basis of a number of FI methods for the determination of nitrate and/or TON. The most commonly used derivatization chemistry involves diazotization of the reduced nitrate and subsequent coupling with sulphanilamide and N-(1-naphthyl)ethylenediamine [48] which has been optimized for field use for a period of 30 days in a remotely deployed FI-based TON monitor [22].

*Table 1. Measured flow rates from pump tube trials over 40 days at room (20 °C) temperature.*

| Time days | Flexible modified PVC ('Tygon Yellow') ml min$^{-1}$ | Flexible PVC ('Tygon') ml min$^{-1}$ | Silicone rubber ml min$^{-1}$ | Butyl rubber ('Ismaprene') ml min$^{-1}$ |
|---|---|---|---|---|
| 3 | 1.0 | 1.0 | 0.7 | 1.2 |
| 5 | 1.0 | 1.0 | 0.8 | 1.1 |
| 20 | 0.9 | 0.9 | 0.8 | 1.0 |
| 33 | 0.9 | 0.9 | 0.7 | 1.0 |
| 40 | 0.9 | 0.9 | 0.8 | 1.0 |

*Table 2. Measured flow rates at constant temperatures over 17 days.*

| Temperature °C | Modified flexible PVC ml min$^{-1}$ | Flexible PVC ml min$^{-1}$ | Silicone rubber ml min$^{-1}$ | Butyl rubber ml min$^{-1}$ |
|---|---|---|---|---|
| 22 | 1.0 | 1.1 | 0.9 | 1.1 |
| 5 | 0.8 | 0.9 | 0.8 | 1.0 |
| 10 | 0.9 | 0.9 | 0.9 | 0.9 |
| 5 | 0.7 | 0.8 | 0.9 | 0.9 |

*Pump tubing and design*

The pump tube test rig consisted of a four-channel peristaltic pump controlled by a time switch set to run the pump 10 min on, 10 min off. Tubes undergoing tests were connected to a manifold which allowed continuous cycling of water from a small reservoir to the test rig. At regular intervals during the test period (40 days), the flow rate of each tube was measured and the results shown in table 1. On completion of the test, each pump tube contact area with the pump roller was visually inspected to assess wear. It was observed that one of the most commonly used tubing materials, silicone, suffered severe deformation and abrasion to such an extent that the tube was almost completely worn through. Silicone tubing was therefore considered unsuitable for extended continuous use. Both the flexible PVC and butyl rubber tubing sustained slight deformation and wear, but the modified flexible PVC tubing showed negligible deformation or abrasion.

Further tests were carried out on new tubes over a 17 day period to determine the effect of temperature on flow rate and wear characteristics, and the results are shown in table 2. There were no visible signs of wear or deformation on either type of flexible PVC tubing or butyl rubber tubing, but a small flat spot had been worn at the tube pump/pump roller contact area on the silicone tubing.

From these tests, it was concluded that although the silicone exhibited more stable flow characteristics, the poor wear resistance made it unsuitable for long term deployment. The butyl rubber tubing was rejected for the same reasons, although the wear characteristics were better than those of silicone rubber. Of the two types of PVC tubing tested, the modified material was considered most suitable for long term deployment in an FI system. There was a significant temperature effect, but this was not considered to be a problem due to stabilization of the FI system temperature when submersed.

During the flowcell development work, pulsing of the output signal was observed and this was attributed to the design of peristaltic pumps used. The original pumps fitted were identical to those used in the terrestrial nutrient monitor developed at the University of Plymouth [15], i.e. 12 V DC eight-roller, single tensioner four-channel pumps, and extended (300 h) use highlighted this deficiency. The cause of the problem was the single tensioner design used, which meant that when different diameter tubes are used, the force required to initiate and sustain flow in the smallest diameter tube was also applied to the larger diameter tube. This effectively applied a 'brake' to the system and excessive lateral force to the pump bearings. The observed effects of this were poor pump start-up and flow initiation, particularly at low temperatures, premature wear of pump bearings and pump motor stalling, causing excessive current drain from the power supply.

The pump problems also had a dramatic effect on the performance of the detection system, particularly at low nitrate concentrations, due to poor signal to noise. It was decided to engineer out the problem rather than compromising the analytical data by applying software smoothing routines. The single tensioner pumps were therefore replaced with 12 V DC, eight-roller, two-channel pumps with separate tube tensioners for each channel. The difference in pump performance from standard peaks obtained with a 0.5 mg l$^{-1}$ Nitrate-N standard is shown in figure 5.

*Integrated system design and operation*

The pressure housing was successfully depth-tested empty to 45 m and the complete system was regularly operated at depths of up to 37 m, the maximum depth available at the test location (Barn Pool in Plymouth Sound), during field trials. These submersed trials showed that the system reached ambient temperature approximately 10 min

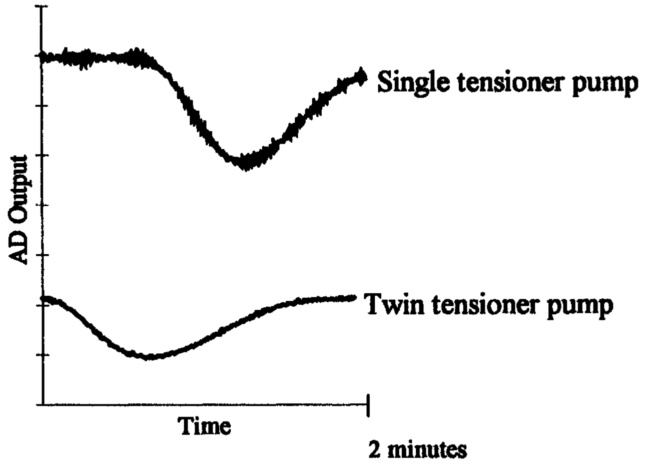

*Figure 5. Detector output using single tensioner and twin tensioner peristaltic pumps for a 0.5 mg l$^{-1}$ Nitrate-N standard.*

after deployment and therefore could be operated without on-board calibration standards if the ambient temperature was known. A temperature sensor was incorporated to allow for deployment in regions of significant temperature fluctuations, e.g. near the surface.

During one submersed deployment, a reagent tube connector failed and the instrument module filled with seawater. The fail-safe mechanism was inadvertently overridden so that the system flooded whilst powered up. The sensor was retrieved and the instrument module immediately flushed with freshwater and dried. The on-board computer and I/O boards were later removed, and thoroughly cleaned and tested to find that an *EPROM* had blown. This was replaced and the system functioned normally, proving that the application of the conformal coating and RTV compounds limits the amount of damage should a leak occur. The outer cage assembly also functioned as designed by protecting the internal components from serious impacts during approximately nine months of field testing.

*Filtration*

The final filtration level of 5 µm was found to be perfectly adequate for the system. This level gave well-defined peaks and protected the reduction column from blockage, whereas early bench testing in estuarine conditions without filtration gave poorly defined flat-bottomed peaks and quickly blocked the reduction column.

*Reagent pack*

Sampling every 10 min during submersed 13 h tidal cycle deployments resulted in a typical carrier and mixed colour reagent consumption of approximately 200 ml and 150 ml, respectively. The sampling routine included a 90 s sample loop flush prior to injection when reagents were continuously pumping. However, the system could be configured to shutdown the reagent pump during the sample loop flush to conserve reagents if required.

*Flowcell*

During the development stage, the results from the prototype 10 mm path length acrylic-bodied flowcell were encouraging, so further examples were constructed with different path lengths to cover different ranges. Flowcells with 5 mm and 20 mm path lengths were constructed, but both were found to be unsatisfactory when tested with the same standards. Output from the 5 mm version was particularly unstable, so no useful results were obtained. The 20 mm version output was stable but response was much lower than expected when compared to the original 10 mm flowcell. This was eventually attributed to internal diffraction caused by the clear acrylic body of the flowcell and the beam angle of the LED.

A series of flowcells was then constructed from opaque rigid PVC and preliminary tests gave much better results, but the wetting properties of the PVC increased the occurrence of trapped air bubbles. A series of modifications was then implemented to alleviate this problem. Flow properties of the flowcell and manifold were eventually improved to a point where air bubbles were not trapped in the system under normal operating conditions. It was also found that the heatsink was unnecessary so the LED was fitted directly into the flowcell body. A flowcell with a 20 mm path length was used for all subsequent deployments unless otherwise stated.

*Reduction column*

During extended operation, carrier flow pushed the reductor material towards the outlet of the glass-bodied copperized-cadmium reduction column. This resulted in a small air pocket being trapped between the front face of the reductor material and the glass wool plug on the inlet side, causing gradual oxidation of the copperized-cadmium from that end. Experience showed, however, that the performance of this type of reduction column remained consistent for at least eight weeks, with no significant deterioration until approximately 30% of the total length of the column was oxidized. A more rapid deterioration was observed when the column packing was disturbed in several places and the larger air pocket at the front face formed smaller air pockets within the reductor material. This usually happened when the column was subjected to severe shock or flow disruptions caused by mechanical problems elsewhere in the system.

Copperized-cadmium wire columns [42, 43] were investigated and were initially found to perform as well as the packed columns. However, their long-term performance was affected if they were not removed from the system and stored correctly when not in use. In both cases, when wire columns were permanently fitted to the instrument, the columns deteriorated to a point where they were not fit for the purpose within two weeks. Column deterioration was accelerated by the systems' inability to maintain the 1 m column full of carrier solution when at rest (a problem that does not occur with packed reduction columns). Oxidation of the wire column was initiated on the areas of uncovered cadmium at the wire to tube wall contact points. The oxidation process proceeded until the majority of the cadmium wire was oxidized and formed a sludge within the tubing.

The flow characteristics of the wire column did not suit the instrument; the time from injection to commencement of data collection was increased from 1.5 min for the packed column to 3 min for the wire column. Additionally, the resultant peak from the wire column was less well defined than that obtained from a packed reduction column. It was therefore decided to concentrate on optimizing and ruggedizing the packed reduction column for use in the submersible sensor. Performance and flow characteristics of the prototype acrylic-bodied reduction column were found to be excellent, and the use of 'Nylon' mesh retainers before and after the reductor eliminated the air bubbles associated with the original glass-bodied reduction column.

*Bench and field tests*

During its development period, the instrument was regularly bench tested alongside a FIAstar 5020 analyser [22] during surveys on the Tamar estuary aboard the PML research vessel RV Tamaris. The cruises com-

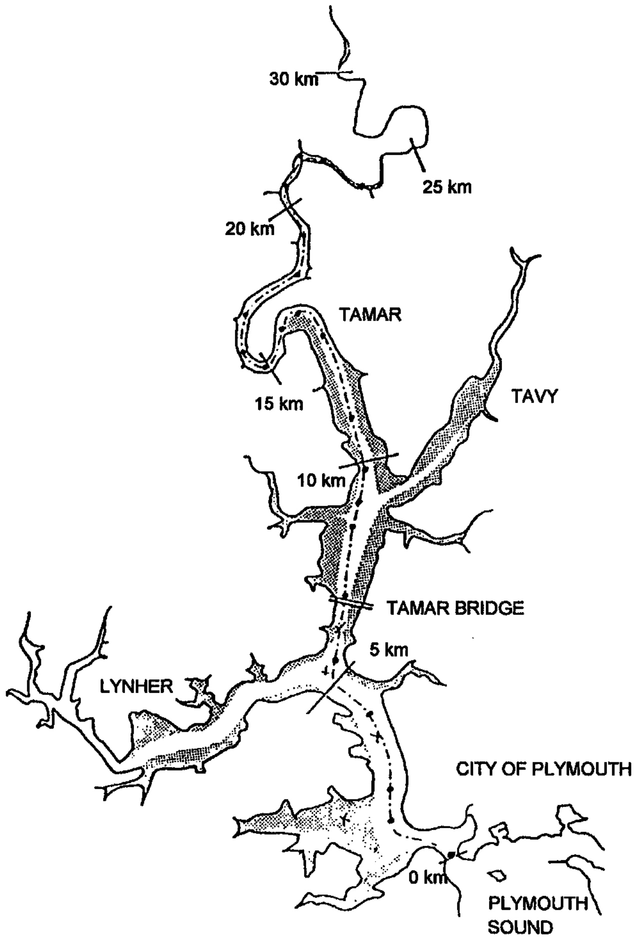

*Figure 6. Map of the Tamar estuary. Sampling points were at Drakes Island (0 km upstream), Skinham Point (8 km upstream), Weir Quay (13 km upstream), Tinnel (15 km upstream), Whitsam (18 km upstream), Museum (21 km upstream) and Calstock (23 km upstream).*

*Table 3. Comparison of field data obtained from the FI nutrient sensor and a FIAstar Analyser during the Tamar survey on 9 November 1994. There was no significant difference based on a t-test at* $P = 0.05$.

| *Sample station* | *Position* | *Distance uptream km* | *FI nutrient sensor* | *FIAstar* |
|---|---|---|---|---|
| | | | *Nitrate-N mg*$^{-1}$ | |
| 1 | Drakes Island | 0 | < range | 0.33 |
| 2 | Skinham Point | 8 | 1.89 | 1.93 |
| 3 | Weir Quay | 13 | > range | 2.79 |
| 4 | Tinnel | 15 | 1.96 | 2.14 |
| 5 | Whitsam | 18 | 1.72 | 1.60 |
| 6 | Museum | 21 | 1.56 | 1.54 |
| 7 | Calstock | 23 | 1.66 | 1.55 |

Sensor configuration: 130 µl sample loop; 20 mm path length flowcell; 5 µm inlet filter.

Conditions: High tide after several days heavy rainfall.

*Table 4. Calibration data and between batch reproducibility for three series of shipboard deployments in the Tamar Estuary.*

| *Nitrate-N mg l*$^{-1}$ | *20 Feb. 1995* | *2 March 1995* | *7 March 1995* | *Mean* | *Rsd %* |
|---|---|---|---|---|---|
| 0.00 | 390 | 350 | 377 | 372 | 5.5 |
| 0.10 | 630 | 673 | 637 | 647 | 2.9 |
| 0.25 | 781 | 784 | 845 | 803 | 4.5 |
| 0.50 | 1442 | 1237 | 1321 | 1333 | 7.7 |
| 0.75 | 1775 | 1817 | 1617 | 1736 | 6.1 |

*Table 5. Results from a low level calibration. Response shown relative to the blank.*

| *Nitrate-N mg l*$^{-1}$ | *n* | *Mean* | *Rsd %* |
|---|---|---|---|
| 0.0000 | 3 | 0 | 0.0 |
| 0.0014 | 17 | 6 | 4.9 |
| 0.014 | 3 | 54 | 3.1 |
| 0.028 | 3 | 110 | 1.5 |
| 0.042 | 3 | 148 | 1.1 |
| 0.056 | 3 | 187 | 0.9 |

menced at Sutton harbour on or around high tide and followed the course of the river to Calstock, pausing to take samples at various points along the cruise track, as shown in figure 6. Analytical performance improved with each deployment to a point where, for a particular range, the instrument produced results in good agreement with those of the 'FIAstar', as shown in table 3. The FIAstar method had previously been validated by participation in two inter-laboratory exercises, for nitrate in river water [EU Community Bureau of Reference (BCR)] and sea water (International Council for Exploration of the Sea), respectively [49]. The between batch reproducibility for three series of shipboard calibrations (0.10–0.75 mg l$^{-1}$ $NO_3$-N) in the Tamar estuary were in the range of 2.9–7.7% rsd, as shown in table 4. These were carried out with the same batch of reagents and the same reduction column over a period of two weeks, with reagent bags filled on the evening prior to the deployment. The within batch reproducibility ($n = 5$) was always <5% rsd. Results from a typical low range calibration (0.0014–0.056 mg l$^{-1}$ $NO_3$-N) are shown in table 5.

*Submersed deployments*

To date, the sensor has successfully operated in the Tamar estuary at depths of up to 37 m on 14 separate deployments of 2–3 h duration in various weather and tidal conditions. Typical results from one such submersed deployment at a depth of 20 m are shown in table 6. A smaller sample volume (130 µl) was used for this particular deployment to increase the linear range. The sensor has also been successfully deployed in the North Sea for three complete tidal cycles (13 h) at 4 m below the surface [50]. Continuous data (every 10–15 min) for a 7.5 h submersed deployment at 53° 35.19′ N, 00° 13.09′ E on 3 June 1995 are given in table 7, together with shipboard AutoAnalyzer data and turbidity and salinity data.

*Table 6. Results from a 20 m submersed deployment at Barn Pool (Plymouth Sound) on 23 March* 1995.

| Time | °C | Submersed FI nutrient sensor | FIAstar |
|---|---|---|---|
| | | Nitrate-N (mg $l^{-1}$) | |
| 1055 | 10.0 | 0.17 | 0.19 |
| 1110 | 10.0 | 0.19 | 0.25 |
| 1135 | 10.0 | 0.22 | 0.26 |
| 1145 | 10.0 | 0.21 | 0.19 |
| 1205 | 11.0 | 0.16 | 0.17 |

High tide was at 1006 and low tide was at 1609.

*Table 7. Results from submersed FI nutrient sensor deployment at 53° 35.19′ N, 00° 13.09′ E on 3 June 1995 together with shipboard AutoAnalyzer® data for $NO_3$-N and turbidity and salinity data.*

| Time | Submersed FI nutrient sensor mg $l^{-1}$ | Shipboard AutoAnalyzer® mg $l^{-1}$ | Turbidity ppm | Salinity |
|---|---|---|---|---|
| 08 55 | 0.050 | — | 2.0 | 33.8 |
| 09 00 | 0.050 | — | — | 33.8 |
| 09 30 | 0.055 | — | 3.0 | 33.8 |
| 09 45 | 0.050 | — | 1.0 | 33.8 |
| 10 00 | 0.050 | — | — | 33.8 |
| 10 15 | 0.045 | — | 3.0 | 33.9 |
| 10 30 | 0.040 | 0.011 | — | 34.1 |
| 10 45 | 0.043 | 0.011 | 3.0 | 34.1 |
| 11 00 | 0.060 | 0.011 | — | 34.1 |
| 11 15 | 0.060 | 0.000 | 3.0 | 34.1 |
| 11 45 | 0.035 | 0.000 | 6.0 | 34.1 |
| 12 00 | 0.045 | 0.000 | — | 34.0 |
| 12 15 | 0.065 | 0.021 | 12.0 | 33.7 |
| 12 30 | 0.090 | 0.102 | — | 33.7 |
| 12 45 | 0.140 | 0.120 | 14.0 | 33.6 |
| 12 55 | 0.155 | 0.154 | — | 33.5 |
| 13 05 | 0.180 | 0.160 | — | 33.5 |
| 13 15 | 0.195 | 0.190 | 19.0 | 33.4 |
| 13 25 | 0.225 | 0.210 | — | 33.3 |
| 13 35 | 0.230 | 0.220 | 20.0 | 33.3 |
| 13 45 | 0.250 | 0.230 | — | 33.3 |
| 13 55 | 0.270 | 0.260 | — | 33.3 |
| 14 05 | 0.270 | 0.260 | — | 33.2 |
| 14 15 | 0.275 | 0.270 | 24.0 | 33.3 |
| 14 25 | 0.270 | 0.290 | — | 33.2 |
| 14 35 | 0.275 | 0.290 | — | 33.2 |
| 14 45 | 0.285 | 0.300 | 29.0 | 33.1 |
| 14 55 | 0.295 | 0.340 | — | 33.1 |
| 15 05 | 0.315 | 0.350 | — | 33.0 |
| 15 15 | 0.320 | 0.360 | 31.0 | 32.9 |
| 15 25 | 0.330 | 0.400 | — | 32.9 |
| 15 35 | 0.335 | 0.410 | — | 32.8 |
| 15 45 | 0.340 | 0.430 | 29.0 | 32.8 |
| 15 55 | 0.350 | 0.460 | — | 32.7 |
| 16 05 | 0.330 | 0.470 | — | 32.7 |
| 16 15 | 0.345 | 0.480 | 28.0 | 32.3 |
| 16 25 | 0.395 | 0.500 | — | 32.2 |
| 16 35 | 0.440 | 0.510 | — | 32.2 |

High tide was at 0930.

### Conclusions

The feasibility of using FI in a submersible nitrate sensor in estuarine and coastal waters has been demonstrated. The reliability of the sensor was good and the analytical performance was more than capable of measuring nitrate concentrations commonly found in these situations. The flexibility of design allowed the sensor to operate in numerous configurations and, with further development, could include additional chemistries, e.g. for the other nutrient elements.

### Acknowledgements

This work was funded by an NERC SIDAL Special Topics Grant GST/02/0669. Current developments are being supported by Chelsea Instruments and the DTI via a EUREKA EUROMAR project (*NUT*rient *S*ensor *for* a *B*enthic *In S*itu *C*aro*U*sel *I*ntegrated European *T*echnology). The authors would like thank A. Hopkins (University of Plymouth) and A. W. Morris, R. J. M. Howland, N. Bloomer and I. Gilson (Plymouth Marine Laboratory) for their support and assistance.

### References

1. Grasshoff, K., Ehrhardt, M. and Kremling, K., 1976, *Methods of Sea Water Analysis* (New York: Verlag Chemie).

2. D. O. E. Pollution Paper 26, 1986, *Nitrate in Water* (London: HMSO).

3. Casey, H. and Clarke, R. T., 1979, *Freshwater Biology*, **9**, 91.

4. Macdonald, R. W. and Mclaughlin, F. A., 1982, *Water Research*, **16**, 95.

5. Clementson, L. A. and Wayte, S. E., 1992, *Water Research*, **26**, 1171.

6. U. S. EPA Method No. 353.2, 1979, *Methods for the Chemical Analysis of Waters and Wastes*, (Washington).

7. Roman, M., Dovi, R., Yoder, R., Dias, F. and Warden, B., 1991, *Journal of Chromatography*, **546**, 341.

8. Maclaurin, P., Worsfold, P. J., Townshend, A., Barnett, N. W. and Crane, M., 1991, *Analyst*, **116**, 710.

9. Luque de Castro, M. D. and Valcarcel, M., 1990, *International Journal of Environmental Analytical Chemistry*, **38**, 171.

10. Trojanowicz, M., Benson, R. L. and Worsfold, P. J., 1991, *Trends in Analytical Chemistry*, **10**, 11.

11. 1990, Flow injection analysis—an essay review and analytical methods. *Methods for the Examination of Waters and Associated Materials* (London: HMSO).

12. 1992, Application Note AN 136/91. (Högenäs, Sweden: Perstorp Analytical).

13. Clinch, J. R., Worsfold, P. J. and Casey, H., 1987, *Analytica Chimica Acta*, **200**, 523.

14. Clinch, J. R., Worsfold, P. J., Casey, H. and Smith, S. M., 1988, *Anaytical Proceedings*, **25**, 71.

15. Blundell, N. J., Casey, H. and Worsfold, P. J., 1993, *Journal of Automatic Chemistry*, **15**, 159.

16. Casey, H., Clarke, R. T., Smith, S. M., Clinch, J. R. and Worsfold, P. J., 1989, *Analytica Chimica Acta*, **227**, 379.

17. Worsfold, P. J., Clinch, J. R. and Casey, H., 1987, *Analytica Chimica Acta*, **197**, 43.

18. Clinch, J. R., Worsfold, P. J. and Sweeting, F., 1988, *Analytica Chimica Acta*, **214**, 401.

19. Benson, R. L., Worsfold, P. J. and Sweeting, F., 1990, *Analytica Chimica Acta*, **238**, 177.

20. Johnson, K. S., Beehler, C. L. and Sakamoto-Arnold, C. M., 1986, *Analytica Chimica Acta*, **179**, 245.

21. Johnson, K. S., Sakamoto-Arnold, C. M. and Beehler, C. L., 1989, *Deep-Sea Research*, **36**, 1407.

22. McCormack, T., David, A. R. J., Worsfold, P. J. and Howland, R. J. M., 1994, *Analytical Proceedings*, **31**, 81.

23. Dasgupta, P. K., Bellamy, H. S., Liu, H., Lopez, J. L., Loree, J. L., Morris, K., Petersen, K. and Kalam, K. M., 1993, *Talanta*, **40**, 53.
24. Military Standard MIL-STD-2000, 1989, Standard requirements for soldered electrical and electronic assemblies.
25. Warygold, J., *How to Select Conformal Coatings for Printed Circuit Boards* (Woodside, New York: Humiseal Div., Columbia Chase Corp. ).
26. Naisbitt, G. K., *An Explanation of Conformal Coatings* (Camberley, UK: Concoat).
27. Montgomery, H. A. C. and Dymcock, J. F., 1962, *Analyst*, **87**, 374.
28. Jenkins, D. and Medsker, L. L., 1964, *Analytical Chemistry*, **36**, 610.
29. Brown and Bellinger, E. G., 1978, *Water Research*, **12**, 223.
30. Mullin, J. B. and Riley, J. P., 1955, *Analytica Chimica Acta*, **12**, 464.
31. Downes, M. T., 1978, *Water Research*, **12**, 673.
32. Chow, J. J. and Johnstone, M. S., 1962, *Analytica Chimica Acta*, **27**, 441.
33. Matsananga, K. and Nishimura, M., 1969, *Analytica Chimica Acta*, **45**, 350.
34. Bajic, S. J. and Jaselskis, B., 1985, *Talanta*, **32**, 115.
35. Margeson, J. H., Suggs, J. C. and Midgett, M. R., 1980, *Analytical Chemistry*, **52**, 1955.
36. Morris, A. W. and Riley, J. P., 1963, *Analytica Chimica Acta*, **29**, 272.
37. Gangush, R. F. and Heath, R. T., 1984, *Water Research*, **18**, 449.
38. Wood, E. D., Armstrong, F. A. J. and Richards, F. A., 1967, *Journal of the Marine Biological Association of the UK*, **47**, 23.
39. Lambert, R. S. and Dubois, R. J., 1977, *Analytical Chem*istry, **47**, 955.
40. Oudot, C. and Montel, Y., 1988, *Marine Chemistry*, **24**, 239.
41. Nydahl, F., 1976, *Talanta*, **23**, 349.
42. Stainton, M. P., 1974, *Analytical Chemistry*, **46**, 1616.
43. 1981, Oxidised nitrogen in waters. *Methods for the Examination of Waters and Associated Materials* (London: HMSO).
44. Willis, R. B., 1980, *Analytical Chemistry*, **52**, 1377.
45. Kouparis, M. A., Walczak, K. M. and Malmstadt, H. V., 1982, *Analytica Chimica Acta*, **142**, 119.
46. Hydes, D. J. and Hill, N. C., 1985, *Estuarine, Coastal and Shelf Science*, **21**, 127.
47. Van Staden, J. F., 1982, *Analytica Chimica Acta*, **138**, 403.
48. Benschneider, K. and Robinson, R. J., 1952, *Journal of Marine Science*, **11**, 87.
49. McCormack, T., 1996, Flow injection chemistries for the in situ monitoring of nutrients in sea water. PhD thesis, University of Plymouth.
50. David, A. R. J., McCormack, T., Morris, A. W. and Worsfold, P. J., 1998, *Analytica Chimica Acta*, **261**, 63.

